# Adjuvanticity of β -Glucan for Vaccine Against *Trichinella spiralis*

**DOI:** 10.3389/fcell.2021.701708

**Published:** 2021-07-12

**Authors:** Yi Liu, Xiaolei Liu, Li Yang, Yangyuan Qiu, Jianda Pang, Xiaoxiang Hu, Zijian Dong, Zengshan Liu, Xuemin Jin

**Affiliations:** Key Laboratory of Zoonosis Research, Ministry of Education, College of Veterinary Medicine, Institute of Zoonosis, Jilin University, Changchun, China

**Keywords:** *Trichinella Spiralis*, β-glucan, adjuvant, dendritic cells, Th1/Th2 response

## Abstract

In the past 30 years, few researches focus on the efficacy of adjuvant against *Trichinella spiralis* infection. Identifying new, improved vaccine adjuvants for *T. spiralis* infection are required. β-glucan are effective and safe as adjuvant for infectious diseases. In this paper, we first observed the adjuvanticity of β-glucan as adjuvant for defensing helminth *T. spiralis in vivo*. We showed that IgG and IgE were elevated in the mice immunized with β-glucan combined with recombinant *T. spiralis* serine protease inhibitor (r*Ts*-Serpin), which is one of the vaccine candidates. Furthermore, *in vitro*, the combination of β-glucan and r*Ts*-Serpin enhanced the maturation of bone marrow dendritic cells (BMDCs) compared to r*Ts*-Serpin alone. We showed that β-glucan + r*Ts*-Serpin –treated BMDCs secreted higher production of IL-12 and IL-10. Moreover, β-glucan + r*Ts*-Serpin –treated BMDCs not only promoted the population of CD4^+^ IFN-γ^+^ T cells, but also enhanced the population of CD4^+^ IL-4^+^ T cells. These findings suggested that β-glucan, as an adjuvant, have the capacity to protect against *T. spiralis* infection via activating both Th1 and Th2 immune response.

## Introduction

Trichinellosis is one of the most common parasitic diseases worldwide in various wild and domestic animals and human (Robertson, 2018). Over the past 30 years, a large number of vaccinations have been undertaken to control *Trichinella spiralis* infection, however, few researches focus on the protection of adjuvant against *T. spiralis* infection (Zhang et al., 2018b). Freund’s adjuvant is efficient but not accepted due to animal welfare. Aluminium-based adjuvants cannot produce enough immunity to the antigens with the application of recombinant subunit vaccine and synthetic vaccine (Temizoz et al., 2016). Thus, there is an urgent need to develop new, improved vaccine adjuvants for the control of *T. spiralis*.

Many polysaccharides are recognized by innate immune cells, thereby regulating immunity in the host (Hou et al., 2016; Zhao et al., 2016; Wattanasiri et al., 2017; Gu et al., 2019). More importantly, many natural polysaccharides are safe with no tissue deposition in the host (Sun et al., 2018). Previously, we showed that lentinan derived from mushrooms can improve the protective immunity of the vaccine on *T. spiralis* infection (Jin et al., 2020b). β-glucans is also present in mushrooms, yeast, oats, barley, seaweed and many other organism species, but does not exist in mammals. It is used as an adjuvant and anti-tumor immunity in vaccines against viral infections as well as immunomodulators in anti-cancer immunotherapy (Borchani et al., 2016). Glucans can stimulate various immune responses, including the production of antibodies, without any negative side effects, and is regarded as a promising immune adjuvant (Cordeiro et al., 2015; Moreno-Mendieta et al., 2017). Up to now, the adjuvanticity of β-glucan on helminth infection such as *T. spiralis* remains undetermined.

Dendritic cells (DCs), the strongest antigen-presenting cell population, are recognized as having unparalleled ability to activate innate and adaptive immune pathways. Adjuvants could activate the mature DCs and have the potential to promote the T cell responses (Saxena and Bhardwaj, 2017), thereby establishing the protection against *T. spiralis* (Coakley and Harris, 2020). The process of DCs maturation includes the secretion of inflammatory cytokines, the increase of MHC class II (MHC-II) cell surface expression, the increase of costimulatory molecules, so that the antigen is presented to the naive T cells (Sato et al., 2017). The immunostimulatory effect of β-glucan and the antigenic protein on DCs are not well described.

Previous studies showed that an antigenic protein, *Ts*-Serpin identified as a vaccine for protecting host against *T. spiralis* (Wu et al., 2009; Xu et al., 2017; Song et al., 2018). Based on this vaccine, in this paper, we evaluated the adjuvanticity of β-glucan in the protection against *T. spiralis in vivo* and *in vitro*.

## Materials and Methods

### Ethics Statement

C57BL/6J mice (female, 4–6 weeks old) were purchased from the Experimental Animal Centre of College of Basic Medical Sciences, Jilin University (Changchun, China) and kept in a temperature-controlled room (22 ± 2°C) under a 12 h dark–light cycle. All animal experiments were performed according to regulations of the Administration of Affairs Concerning Experimental Animals in China. The protocol was approved by the Institutional Animal Care and Use Committee of Jilin University (Permit No. 20170318).

### Generation and Maintenance of *T. spiralis*

The *T. spiralis* isolate (ISS534), genotyped and proved by OIE Collaborating Center on Foodborne Parasites in Asian-Pacific Region, was preserved by serial passages in Wistar rats as described previously (Jin et al., 2020b). Briefly, Wistar rats were orally infected with 3000 infective larvae, and *T. spiralis* muscle larvae were recovered at 35 days post infection (dpi) *via* artificial digestion with pepsin-HCl (1% pepsin and 1% HCl at 37°C for 2 h).

### Preparation of Recombinant *Ts*-Serpin (r*Ts*-Serpin)

Recombinant *Ts*-Serpin (r*Ts*-Serpin) was expressed in *Escherichia coli* (BL21) and purified as previously described (Xu et al., 2017; Jin et al., 2020b). The contaminated endotoxin was effectively removed by ToxOut High Capacity Endotoxin Removal Kit (Biovision, United States), approximately equivalent to 20 pg/mg endotoxin in r*Ts*-Serpin (Jin et al., 2020b).

### Immunization and Challenge Infection

To determine the adjuvanticity of the β-glucan, female C57BL/6J mice were randomly divided into four groups (*n* = 20): (1) control group mice (immunized with PBS only), (2) mice immunized with 50 μg of r*Ts*-Serpin, (3) mice immunized with r*Ts*-Serpin emulsified with Freund’s adjuvants (FCA/FIA) (St. Louis, Mo, United States), (4) mice immunized with r*Ts*-Serpin emulsified with 200 μg of β-glucan in PBS. β-glucan (No. G6513) from barley was purchased from Sigma-Aldrich. The purity of β-glucan was >95% determined by high performance liquid chromatography. Immunization was performed subcutaneously 3 times at 2 week interval. 2 weeks after the final vaccination, all mice were orally infected with 500 *T. spiralis* muscle larvae/mouse.

### Helminth Burden

Intestinal adult worms were collected at 7 dpi, and muscle larvae were recovered and counted at 35 dpi as previously described (Cui et al., 2019). The helminth burden and the percent of reduction in the mean number of adult worms or the recovered muscle larvae per gram (LPG) of muscle by artificially digesting the carcasses were calculated.

### Antibody Determination

Specific antibodies against r*Ts*-Serpin were evaluated at 6 weeks post vaccination (wpv). Blood was collected from mice at 2, 4, and 6 wpv. The titers of anti- r*Ts*-Serpin IgG, IgG1, IgG2a subclasses, and IgE were measured using an indirect enzyme-linked immunosorbent assay (ELISA) as described previously (Jin et al., 2020b).

### Cytokine Production From Spleens

Cytokine production from splenocyte culture supernatants was tested as described previously (Jin et al., 2020b). Briefly, 1 week after the final immunization, CD4^+^ T cells in spleens derived from mice were purified using anti-CD4 magnetic beads (Miltenyi Biotec). The purified CD4^+^ T cells had >90% purity. The CD4^+^ T cells were cultured to 1 × 10^6^ cells/mL in complete RPMI-1640 containing 10% fetal bovine serum (FBS), penicillin (100 U/mL) and streptomycin (100 μg/mL) and treated with r*Ts*-Serpin at a concentration of 20 μg/mL at 37°C for 72 h. The supernatants of CD4^+^ T cells were collected for determining the levels of IFN-γ and IL-4 by ELISA (R&D Systems).

### Isolation and Stimulation of Dendritic Cells

Bone marrow-dendritic cells (BMDCs) were isolated from mouse bone marrow cells as previously described (Jin et al., 2019a). Briefly, bone marrow cells were isolated and cultured in RPMI 1640 medium containing 20 ng/mL recombinant GM-CSF (Sigma–Aldrich), 20 ng/mL IL-4 (Sigma–Aldrich) and 10% FBS at 37°C and 5% CO_2_. Immature DCs were collected on day 7 for further experiments. The DCs were treated with r*Ts*-Serpin (10 μg/mL) alone or combination of r*Ts*-Serpin (10 μg/mL) and β-glucan (50 μg/mL) *in vitro* for 24 h. Dendritic cells were treated with sterile PBS as a control. Cytokines (IL-12p70 and IL-10) levels in the supernatant were quantified by ELISA (R&D Systems). The stimulated DCs were stained with a FITC-conjugated monoclonal antibody (mAb) to CD11c, APC-conjugated mAbs to CD86 (Biolegend, United States) and PE-conjugated mAbs to MHC-II (Biolegend, United States). The cells were analyzed by using a BD FACSCalibur Flow Cytometer and FlowJo software (Tree star Inc, Ashland, OR) (Jin et al., 2020a).

### Co-culture of BMDCs With CD4^+^ T Cells *in vitro*

Spleen CD4^+^ T cells derived from OT-II mice were purified using anti-CD4 magnetic beads (Miltenyi Biotec) as previously described (Jin et al., 2019b). The purified CD4^+^ T cells had >90% purity. DCs (1 × 10^5^/well) and CD4^+^ T cells (1 × 10^6^/well) were cocultured for 72 h with OVA (1 mg/mL). To determine the cytokine production, cells were stimulated with 10 mg/mL Brefeldin A (eBioscience), 50 ng/mL phorbol 12-myristate 13-acetate (PMA) (eBioscience), and 750 ng/mL Ionomycin (eBioscience) for 6 h at 37°C. Cells were stained with FITC-anti-CD4 antibodies (BD Biosciences) for 35 min at 4°C. These cells were fixed, permeabilized using a FIX/PERM set (Biolegend) and blocked in 5% rat serum for 10 min at room temperature in the dark prior to intracellular staining with APC-conjugated mAbs to IFN-γ and PE-conjugated mAbs to IL-4 (Jin et al., 2020a).

To determine CD4 + T-cell proliferation induced by DCs, CD4 + T cells (5 × 10^5^/well) were stained with 5-and 6-carboxyfluorescein diacetate succinimidyl ester (CFSE) (eBioscience) before co-culture with DCs. Samples were analyzed using a BD FACS Calibur Flow Cytometer and FlowJo software (Tree star Inc, Ashland, OR) (Jin et al., 2019b).

### Statistical Analysis

All results are expressed as the mean ± SD. Statistical analysis was performed using the GraphPad Prism 8 software for Windows. One-way, two-way analysis of variance (ANOVA) and independent exponent *t*-test were used to compare the means and determine statistically significant differences between different conditions. *P* values are expressed as ^∗^*P* < 0.05, ^∗∗^*P* < 0.01, and ^∗∗∗^*P* < 0.001.

## Results

### β-Glucan Improved the Immune Protection of Vaccine Against *T. spiralis*

**FIGURE 1:**
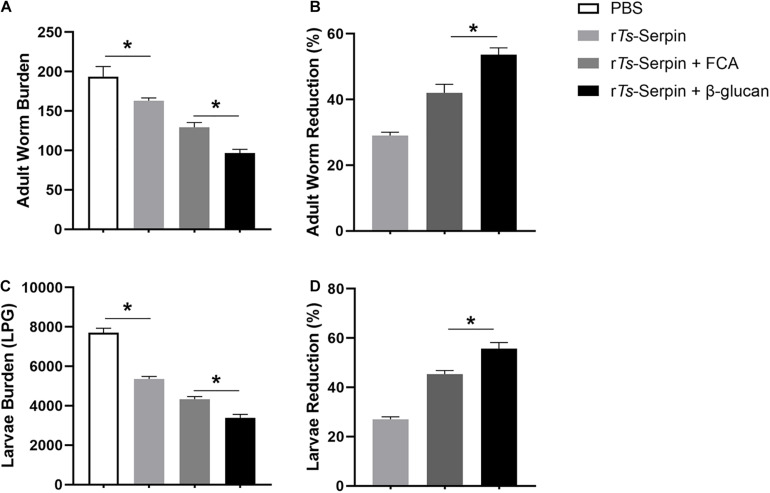
Helminth burden in the immunized mice. **(A)** The number of adults recovered from intestines from immunized mice after challenge with 500 ML of *T. spiralis*. **(B)** The reduction rates of adult worms were analyzed based on the mean number of adult worms. **(C)** The number of muscle larvae (ML) per gram (LPG) in skeletal muscles from immunized mice after challenge with 500 ML of *T. spiralis*. **(D)** The reduction rates muscle larvae were analyzed based on the mean number of recovered muscle larvae per gram (LPG) of muscle from vaccinated groups compared with PBS group. Results are expressed as the mean ± SD of 10 mice per group. The data shown are representative of three independent experiments. *P < 0.05 as indicated by the line (Tukey multiple comparison following ANOVA).

To explore the effect of β-glucan as adjuvant against *T. spralis* infection, combination of β-glucan and r*Ts*-Serpin was administered prior to *T. spralis* challenge. We analyzed the adult worm burden at 7 dpi and muscle larvae burden at 35 dpi. r*Ts*-Serpin significantly reduced the helminth burden compared to the PBS group. Compared with mice from PBS or r*Ts*-Serpin group, immunization could lead to reduced adult worm burden and muscle larvae burden in the mice from FCA + r*Ts*-Serpin group and β-glucan + r*Ts*-Serpin group. And β-glucan + r*Ts*-Serpin significantly decreased the helminth burden compared with FCA + r*Ts*-Serpin (Figures 1A,C). Our results demonstrated that the reduction rate of β-glucan + r*Ts*-Serpin was significantly higher than FCA + r*Ts*-Serpin (Figures1B,D).

### β-Glucan Upregulated the Levels of Specific Antibodies and the Production of Th1/Th2 Cytokines

**FIGURE 2:**
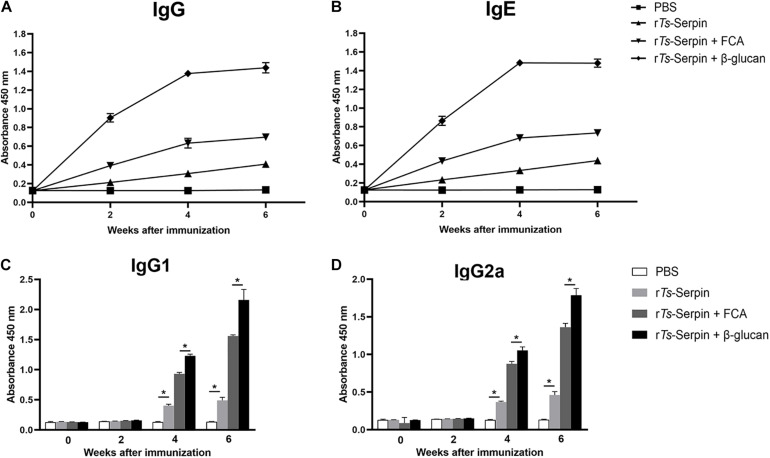
Analysis of humoral immune responses. **(A)** The levels of IgG in the serum were measured by ELISA. **(B)** The levels of IgE in the serum were measured by ELISA. **(C)** The levels of IgG1 in the serum were measured by ELISA at different time points. **(D)** The levels of IgG2a in the serum were measured by ELISA at different time points. The values shown for each group are the mean + SD of the antibody levels (*n* = 10) from three individual experiments *P < 0.05 as indicated by the line (one-way ANOVA with Tukey’s post-test).

To test humoral antibody responses to β-glucan in the host, the levels of IgG and IgE were measured by ELISA. After the second immunization, ELISA results showed the significant enhancement in total IgG level in the mice from β-glucan + r*Ts*-Serpin group, compared with FCA + r*Ts*-Serpin group (Figure 2A). Combination of β-glucan + r*Ts*-Serpin induced elevated levels of IgG1 and IgG2a, compared with FCA + r*Ts*-Serpin group (Figures 2C,D). The levels of specific IgE were also significantly increased in the mice from β-glucan + r*Ts*-Serpin group than FCA + r*Ts*-Serpin group (Figure 2B).

**FIGURE 3:**
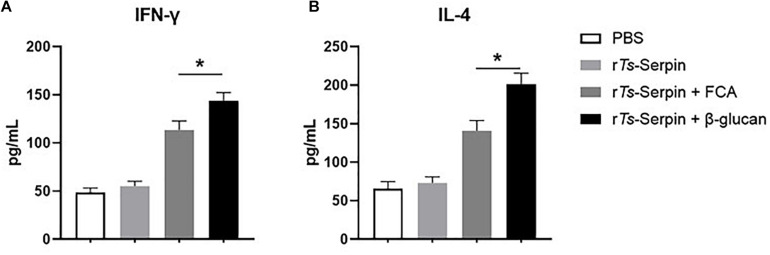
Analysis of cytokine production from CD4^+^ T cells. **(A)** The level of IFN-γ was measured by ELISA one week after the final immunization. **(B)** The level of IL-4 was measured by ELISA one week after the final immunization. The data are the mean ± SD of each group (*n* = 10) from three independent experiments. *P < 0.05 as indicated by the lines.

Furthermore, to confirm whether Th1/Th2-mixed response was induced by administration with β-glucan, levels of Th1/Th2 cytokines, including IFN-γ and IL-4, were detected. Compared with FCA + r*Ts*-Serpin group, elevated production of IFN-γ and IL-4 were observed in the mice from β-glucan + r*Ts*-Serpin group (Figure 3), indicating thatβ-glucan induced a stronger Th1/Th2-mixed response based on the vaccine.

### β-Glucan + r*Ts*-Serpin Regulated the Phenotype of DCs

**FIGURE 4:**
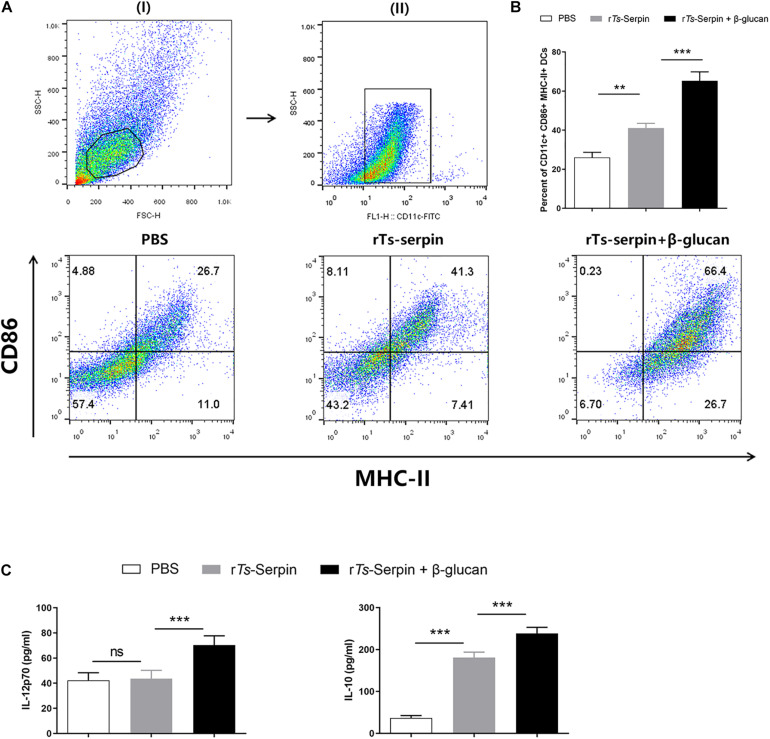
DC phenotype induced by β-glucan + rTs-Serpin. Immature DCs were treated with r*Ts*-Serpin (10 μg/mL) or combination of r*Ts*-Serpin and β-glucan (50 μg/mL) *in vitro* for 24 h. **(A)** (I) gating on viable cells and (II) gating on CD11c^+^ cells. **(B)** Expression of CD11c^+^ CD86^+^ MHC-II^+^ cells were measured. **(C)** Cytokines (IL-12p70 and IL-10) levels in the supernatant were quantified by ELISA Data represent mean ± SD deviations (*n* = 3) of the results from three individual experiments *P < 0.05, **P < 0.01, and ***P < 0.001 vs. the control groups.

An important way of adjuvant is to prolong the antigen exposure and induce the maturation of DCs (Ho et al., 2018). r*Ts*-Serpin significantly enhanced the population of CD11c^+^ CD86^+^ MHC-II^+^ DCs compared to the PBS. We also showed that β-glucan + r*Ts*-Serpin induced the expansion of CD11c^+^ CD86^+^ MHC-II^+^ DCs compared to the PBS or r*Ts*-Serpin (Figures 4A,B). Moreover, r*Ts*-Serpin significantly promoted the level of IL-10, but not IL-12p70. Combination of β-glucan + r*Ts*-Serpin could induce higher levels of IL-12p70 and IL10 compared to r*Ts*-Serpin (Figure 4C).

### β-Glucan + r*Ts*-Serpin –Treated DCs Promoted the Population of Th1/Th2 Cytokines and the Proliferation of CD4^+^ T Cells

**FIGURE 5:**
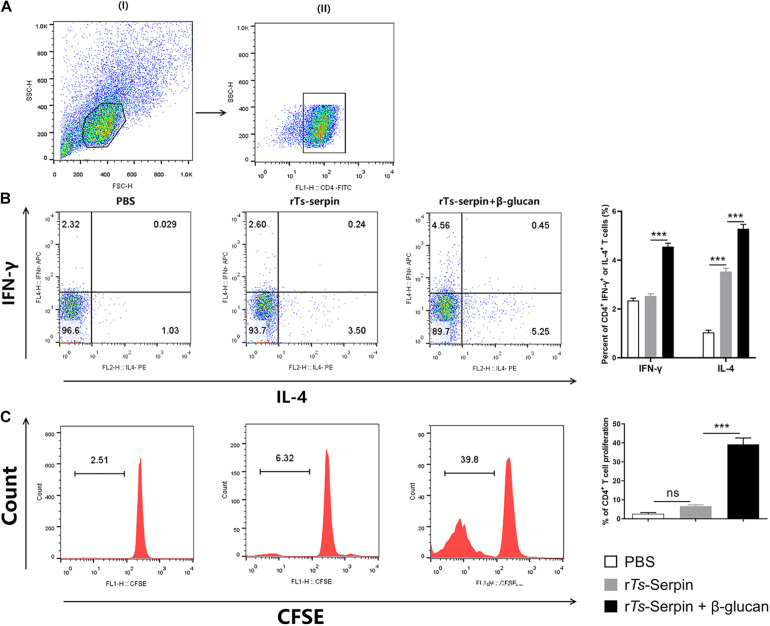
CD4 + T cells response induced by β-glucan + rTs-Serpin –treated DCs. The purity of CD4^+^ T cells were analysis by FACS after magnetic sorting using anti-CD4 magnetic beads. The purified CD4^+^ T cells had > 90% purity. DCs (1 × 10^5^/well) and CD4^+^ T cells (1 × 10^6^/well) were cocultured for 72 h with OVA (1 mg/mL), then cells were incubated with 10 mg/mL Brefeldin A, 50 ng/mL phorbol 12-myristate 13-acetate (PMA) and 750 ng/mL Ionomycin for 6 h at 37°C. **(A)** (I) gating on viable cells and (II) gating on CD4^+^cells. **(B)** Percentage of CD4^+^ IFN-γ^+^ and CD4^+^ IL-4^+^ T cells were determined by flow cytometry. **(C)** CD4 + T cells were stained with CFSE before co-culture with DCs. The proliferation of CD4 + T cells were determined by flow cytometry. Results are shown as mean ± SD (*n* = 3) of three different experiments. *P < 0.05, **P < 0.01, ***P < 0.001 as indicated by line (one-way ANOVA with Tukey’s post test).

It is critical that vaccines contain adjuvants that induce strong T cell proliferation and immune response (Jin et al., 2018). We observed that r*Ts*-Serpin –treated DCs significantly increased the population of CD4^+^ IL-4^+^ T cells compared to PBS group. However, there is no significant difference in the levels of CD4^+^ IFN-γ^+^ T cells. Notably, β-glucan + r*Ts*-Serpin –treated DCs significantly promoted these two different type T cells compared to r*Ts*-Serpin –treated DCs (Figures 5A,B). Moreover, we demonstrated that the proliferation of CFSE –labeled CD4^+^ T cells induced by r*Ts*-Serpin –treated DCs was not increased significantly compared to PBS –treated DCs. FACS results showed that β-glucan + r*Ts*-Serpin –pulsed DCs boosted the proliferation of CD4^+^ T cells compared to DCs treated with r*Ts*-Serpin alone (Figure 5C).

## Discussion

*Trichinella spiralis* causes a huge economic burden to animal husbandry (Bai et al., 2017). Most vaccine trials are conducted in the host generally with FCA (Zhang et al., 2018b), but which is unacceptable due to the toxicity of FCA, which can cause animal pain and damage to meat quality. Oil based adjuvants are widely used in veterinary vaccines, but the host displays the local and systemic reactions (Aucouturier et al., 2001). In addition, aluminium-based adjuvants have the tolerability in the host, however, excessive level of aluminum can lead to reduced renal function, affecting neurological syndromes and dialysis-related dementia (Petrovsky and Aguilar, 2004). We aimed to explore the effect of a novel adjuvant on helminth infection.

Adjuvants based on polysaccharide have the characteristics of low toxicity and safety (Liu et al., 2016; Wattanasiri et al., 2017; Sun et al., 2018). β-glucan are glucose polymers found from yeast cells and bacteria as well (Chan et al., 2009). A high dose up to 10 mg/kg is well tolerated *in vivo*, and no adverse reactions have been seen, which proves that β-glucan is non-toxic (Zhang et al., 2018a). Previously, we found that immunization of β-glucan alone could not reduce the burden of *T. spiralis* (data not shown). However, it has been reported that β-glucan is a powerful adjuvant for favor in antiviral immunity (Soares et al., 2019). However, adjuvanticity of β-glucan against helminth infection is still unknown. *T. spiralis* serine protease inhibitor is likely the potential vaccine target against *T. spiralis* (Song et al., 2018). Our data first demonstrated that β-glucan promoted the vaccine -triggered host defense against *T. spiralis* infection than FCA through upregulating the levels of specific IgG and IgE. Recently it was proposed that β-glucan enhance immunological memory following initial infectious exposure and may provide protection against reinfection (Domínguez-Andrés et al., 2019). Many studies proposed the term “trained immunity” for the enhanced state of innate cells by β-glucan, leading to increased resistance to infection (Netea et al., 2011). Further studies will focus on the role of trained immunity in β-glucan –induced immunoprotecion against *T. spiralis* infection.

Cellular immunity was important for protective immunity. In our study, β-glucan administration could induce immune response that involved both Th1 (IFN-γ) and Th2 (IL-4) cytokines *in vivo*, as other research has shown (Liu et al., 2011). Glycans have been proven to play an important role in the induction of Th2 immune response by *T. spiralis in vivo* (Cvetkovic et al., 2014). We showed that β-glucan triggered a mixed IgG1 (Th2)/IgG2a (Th1) antibody response. Delayed clearance of *T. spiralis* exists in mice deficient IL-4 deficient mice (Scales et al., 2007). Moreover, it was proved that decreased burden of muscle larvae is associated with higher IFN-γ level (Helmby and Grencis, 2003), which could enhance the cytotoxic killing effect of eosinophils, granulocytes and activated macrophages, and exert its protective effect against *T. sprialis* (Yang et al., 2019).

Dendritic cells (DCs) have the ability to regulate naïve T cells responses (Zhu et al., 2010). An ideal adjuvant can induce the generation of DC –mediated immune response through modulation of the phenotype of DCs. We showed that β-glucan administration with recombinant protein led to activation of mature DCs characterized by higher expressions of CD86 and MHC-II, which could trigger T cell proliferation. Our results demonstrated that β-glucan -treated DCs have shown a remarkable capacity for inducing proliferation of CD4^+^ T cells. It has been found that β-glucan also up-regulated CD4^+^ T cell level *in vivo* (Zou et al., 2019). Furthermore, we observed lower production of IL-12 and elevated levels of IL-10 secreted by r*Ts*-Serpin -treated-DCs. IL-10 by DCs can promote the development of Th2 cells (Williams et al., 2013). As expected, these DCs induced strong Th2 immune response, but not Th1 immune response. Interestingly, combination of β-glucan and r*Ts*-Serpin not only promoted CD4^+^ T cells proliferation, but also stimulated a mixed higher levels of Th1 and Th2 immune responses. In other study, β-glucan also showed an excellent adjuvant effect on H5N1 vaccine via promoting the production of Th1 and Th2 related cytokines (Wang et al., 2016).

## Conclusion

We demonstrated that β-glucan significantly improved the efficacy of the vaccine against *T. spiralis* infection *in vivo*. And β-glucan induced mature DCs and modulated the cytokine production by DCs, thereby resulting in the proliferation of CD4^+^ T cells and expansion of mix Th1/Th2 immune response *in vitro*. Thus, β-glucan could be used as an effective immune adjuvant for a vaccine against *T. spiralis*.

## Data Availability Statement

The original contributions presented in the study are included in the article/supplementary material, further inquiries can be directed to the corresponding authors.

## Ethics Statement

The animal study was reviewed and approved by the C57BL/6J mice (female, 4–6 weeks old) were purchased from the Experimental Animal Centre of College of Basic Medical Sciences, Jilin University (Changchun, China) and kept in a temperature-controlled room (22 ± 2°C) under a 12 h dark–light cycle. All animal experiments were performed according to regulations of the Administration of Affairs Concerning Experimental Animals in China. The protocol was approved by the Institutional Animal Care and Use Committee of Jilin University (20170318).

## Author Contributions

YL contributed to article writing, literature search, results evaluation and contributed to literature search and results evaluation. LY performed histologic analysis and article revision. YQ, JP, and ZD performed the final revision of the article and expert opinions. ZL and XL performed the final revision of the article and results evaluation. ZL and XJ contributed to study design. All authors contributed to the article and approved the submitted version.

## Conflict of Interest

The authors declare that the research was conducted in the absence of any commercial or financial relationships that could be construed as a potential conflict of interest.
